# Serum N-glycome biomarker for monitoring development of DENA-induced hepatocellular carcinoma in rat

**DOI:** 10.1186/1476-4598-9-215

**Published:** 2010-08-12

**Authors:** Meng Fang, Sylviane Dewaele, Yun-peng Zhao, Peter Stärkel, Valerie Vanhooren, Yue-ming Chen, Xin Ji, Ming Luo, Bao-mu Sun, Yves Horsmans, Anne Dell, Stuart M Haslam, Paola Grassi, Claude Libert, Chun-fang Gao, Cuiying Chitty Chen

**Affiliations:** 1 Department of Laboratory Medicine, Eastern Hepatobiliary Hospital, Second Military Medical University, 200438 Shanghai, China; 2 Department for Molecular Biomedical Research, VIB, Technologiepark 927, B-9052 Gent-Zwijnaarde, Belgium; 3 Department of Biomedical Molecular Biology, Ghent University, Technologiepark 927, B-9052 Gent-Zwijnaarde, Belgium; 4 Department of Laboratory Medicine, The First People's Hospital of Hangzhou, 310006 Zhejiang, China; 5 Department of Integrated Traditional and Western Medicine, Eastern Hepatobiliary Hospital, Second Military Medical University, 200438 Shanghai, China; 6 Laboratory of Gastroenterology, St. Luc University Hospital, Université Catholique de Louvain, 1200 Brussels, Belgium; 7 Department of Gastroenterology, St. Luc University Hospital, Université Catholique de Louvain, Av. Hippocrate 10, 1200 Brussels, Belgium; 8 Division of Molecular Biosciences,Faculty of Natural Sciences, Imperial College London SW7 2AZ, UK

## Abstract

**Background:**

There is a demand for serum markers for the routine assessment of the progression of liver cancer. We previously found that serum N-linked sugar chains are altered in hepatocellular carcinoma (HCC). Here, we studied glycomic alterations during development of HCC in a rat model.

**Results:**

Rat HCC was induced by the hepatocarcinogen, diethylnitrosamine (DENA). N-glycans were profiled using the DSA-FACE technique developed in our laboratory.

In comparison with control rats, DENA rats showed a gradual but significant increase in two glycans (R5a and R5b) in serum total N-glycans during progression of liver cirrhosis and cancer, and a decrease in a biantennary glycan (P5). The log of the ratio of R5a to P1 (NGA2F) and R5b to P1 [log(R5a/P1) and log(R5b/P1)] were significantly (p < 0.0001) elevated in HCC rats, but not in rats with cirrhosis or fibrosis or in control rats. We thus propose a GlycoTest model using the above-mentioned serum glycan markers to monitor the progression of cirrhosis and HCC in the DENA-treated rat model. When DENA-treated rats were subsequently treated with farnesylthiosalicyclic acid, an anticancer drug, progression to HCC was prevented and GlycoTest markers (P5, R5a and R5b) reverted towards non-DENA levels, and the HCC-specific markers, log(R5a/P1) and log(R5b/P1), normalized completely. **Conclusions**: We found an increase in core-α-1,6-fucosylated glycoproteins in serum and liver of rats with HCC, which demonstrates that fucosylation is altered during progression of HCC. Our GlycoTest model can be used to monitor progression of HCC and to follow up treatment of liver tumors in the DENA rat. This GlycoTest model is particularly important because a rapid non-invasive diagnostic procedure for tumour progression in this rat model would greatly facilitate the search for anticancer drugs.

## Background

Hepatocellular carcinoma (HCC) is the fifth most common cancer and the third leading cause of cancer-related death in the world [1]. Infection with hepatitis B virus (HBV) or hepatitis C virus (HCV) is the major etiologic factor for HCC [2]. A strong correlation exists between cirrhosis and hepatocarcinogenesis, and most patients with HCC have pre-existing cirrhosis. Chronic hepatitis virus infection commonly leads to fibrosis followed by cirrhosis and finally hepatocellular carcinoma. The diagnosis and certainly the follow-up of liver diseases such as cirrhosis and HCC remains a heavily debated problem [3-6]. Alpha-fetoprotein (AFP) is commonly used as a serum biomarker, but its level is normal in one-third of HCC patients [7,8]. The gold standard for diagnosis of HCC is histopathological examination of liver biopsy. Thus, a sensitive and specific non-invasive serological marker is needed for the early diagnosis of HCC and for monitoring its treatment.

Abnormal protein glycosylation is associated with malignant transformation of cells. The N-linked sugar chains are altered in various tumors, and certain glycan structures are well-known markers of tumor progression [9-12]. Most serum N-linked glycoproteins are synthesized by the liver or by B-lymphocytes, and any changes in serum total N-glycans could reflect an alteration of liver or B-lymphocyte physiology. Thus, changes in the quantity and type of N-glycans in serum could be utilized for the non-invasive diagnosis of liver diseases. We and others have reported that N-glycan profiles generated by DSA-FACE (DNA Sequencer Assisted - Fluorophore Assisted Carbohydrate Electrophoresis) could be used as a non-invasive marker for HCV-related liver cirrhosis [13,14], HBV-related fibrosis [15], and HBV-related HCC [16,17]. Our previous study also showed that combining serum N-glycan markers with AFP improves the efficacy of diagnosing HBV-related HCC [18].

Investigations of hepatocarcinogenesis make use of animal models. HCC can be induced in laboratory animals by various chemicals, such as aflatoxin B1, 2-acetylaminofluorene (2-AAF) and diethylnitrosamine (DENA) [19-21]. In this study, we injected rats with DENA, which causes the sequence of fibrosis, cirrhosis and HCC. By monitoring N-glycomic profiles after DENA injection, we identified serum N-glycan biomarkers that can be used to monitor liver fibrosis, cirrhosis and HCC. These serum glycan markers were validated in a rat model in which tumor development is prevented by using an antitumor drug (S-trans-trans-farnesylthiosalicyclic acid; FTS).

## Results

### Alteration of the Profiles of Serum Protein N-glycans during the development of HCC

**Figure 1:**
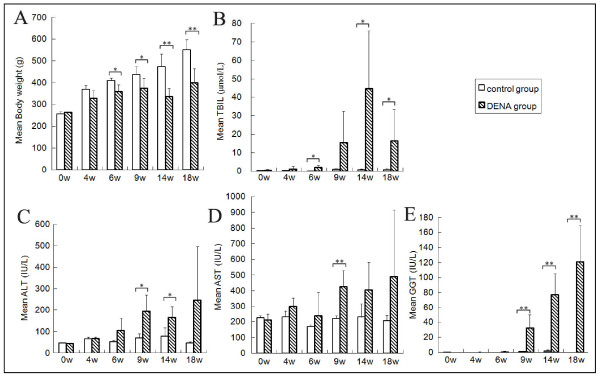
**Body weight and biochemical tests at different time points after DENA administration (weeks)**. (A) Body weight (g); (B) serum total bilirubin (TBil, μmol/L); (C) serum alanine aminotransferase (ALT, IU/L); (D) serum aspartate aminotransferase (AST, IU/L); and (E) serum γ-glutamyltransferase (GGT, IU/L). All the values are expressed as mean ± SD on the vertical axis. "Open square" represents the control group and "slash square" represents the DENA group. Asterisks indicate statistically significant differences between the groups (* *p *< 0.05, **p < 0.01 and ***p < 0.001).

**Figure 2:**
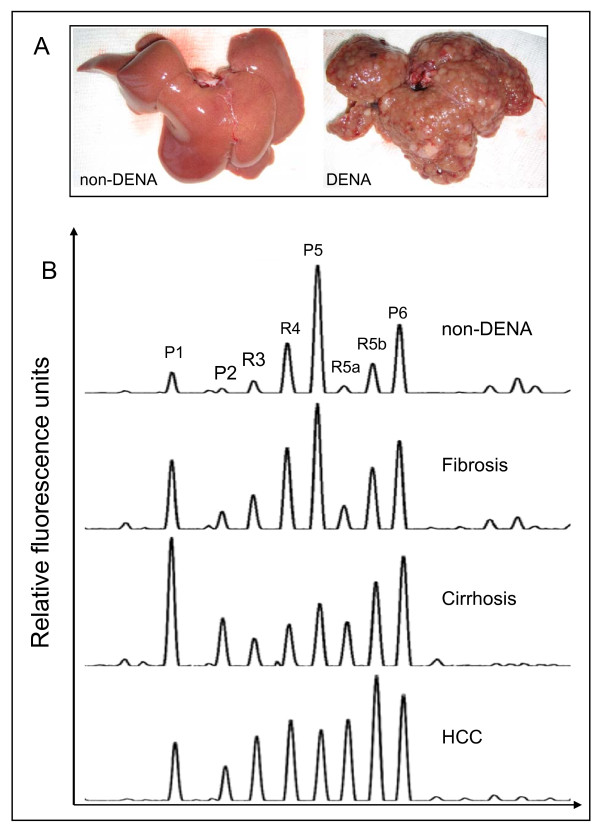
**Representative serum desialylated N-glycan profiles in DENA rats**. (A) Liver of untreated control rat (left) and liver of DENA treated rat showing multifocal HCC nodules. (B) The four panels (from top to bottom) are typical serum N-glycan fingerprints from control, fibrotic, cirrhotic and HCC rats. Eight major glycan peaks were detected in rat serum, and their patterns in the four rat groups showed considerable differences. The vertical axis represents the glycan values of the peaks as percent relative fluorescence level. The X-axis represents the retention time of N-glycans.

We induced liver tumors in Sprague-Dawley rats by injecting DENA intraperitoneally three times a week for 14 weeks. Starting six weeks after initiation of DENA treatment, the body weight (BW) of DENA-injected rats became significantly lower (p < 0.05) than that of control rats (Figure 1A). Administration of DENA also led to an increase in serum Tbil, AST, ALT and GGT, which indicates liver damage (Figure 1B-E). To monitor tumor development, the rats were sacrificed at different times during the DENA administration phase. Histological examination indicated the evolution of liver disease caused by hepatotoxicity of DENA (Table 1). Rats in the DENA group developed mild liver fibrosis (F1-F2) during week 4 and severe fibrosis (F3) and cirrhosis (F4) during week 9. HCC nodules arose from cirrhotic livers after 14 weeks of DENA administration. By week 18, all rats in the DENA group had developed diffusive HCC in cirrhotic livers (Figure 2A).

**Table 1:** Histological diagnosis of liver tissue from rats sacrificed at different time point

DENA Administration	Group	Histological diagnosis (no. animals)				
		
		Healthy	Fibrosis stage			HCC
				
			F1-2	F3	F4 (cirrhosis)	
	control	3				
	
week 0	DENA	2				

	control	3				
	
week 4	DENA		6			

	control	3				
	
week 6	DENA		12			

	control	3				
	
week 9	DENA			11	4	

	control	3				
	
week 14	DENA				5	2

	control	6				
	
week 18	DENA					13

**Figure 3:**
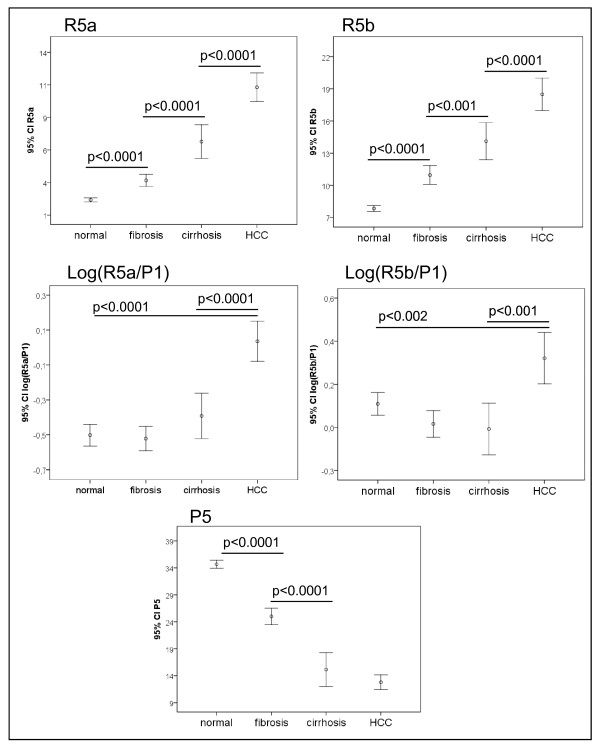
**N-glycan values that differ significantly between control, fibrotic, cirrhotic and HCC groups**. The vertical axis represents the glycan values of GycoTest markers R5a, R5b, log(R5a/P1), log(R5b/P1) and P5. Error bars represent 95% confidence intervals for means. Statistical significance of differences between groups is indicated by the p value.

**Figure 4:**
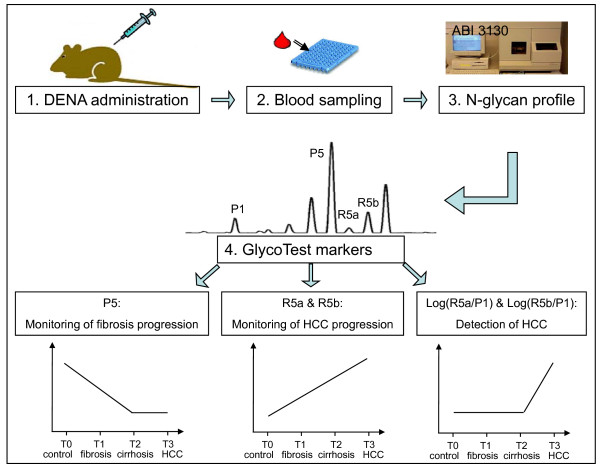
**A scheme for using the GlycoTest model to monitor progression of HCC in DENA rats**. The GlyoTest model includes the following steps: (1) DENA administration, (2) blood sampling during progression of HCC, (3) serum N-glycan profiling using DSA-FACE, and (4) analysis of GlycoTest markers. The GlycoTest markers are as follows: a decrease in P5 indicates fibrosis progression; an increase in R5a and R5b indicates HCC progression; and an elevation of log(R5a/P1) and log(5Rb/P1) points to development of HCC.

We analyzed the serum N-glycan profiles in DENA-treated rats and control rats using DSA-FACE. The serum desialylated N-glycan profiles (represented as peaks) gradually changed during progression of HCC in DENA-treated rats, but they remained unchanged in the control rats (Figure 2B). We quantified each peak by normalizing its height to the sum of the heights of all peaks in the profile, and then we statistically compared the peaks of control (n = 23), fibrotic (n = 29), cirrhotic (n = 9) and HCC-bearing (n = 15) rats. The intra- and inter-assay coefficients of variations (CVs) of the glycan analysis are less than 5%. All main peaks were altered in DENA-treated animals compared to controls (Additional file 1, Figure S1). Peaks R5a and R5b increased gradually but significantly (p < 0.05) during development of fibrosis and HCC in the treated rats (Figure 3), which suggests that R5a and R5b could be used as biomarkers for monitoring progression of fibrosis and HCC in the DENA rat model. Interestingly, P1 and P2 gradually increased in the DENA rats as fibrosis progressed (Additional file 1, Figure S1). However, their levels were significantly lower in HCC rats than in cirrhotic rats. To distinguish HCC from cirrhosis, we need a marker that changes only in HCC but not in cirrhosis. Moreover, for precision and accuracy, a ratio of two variables is better than the use of a single measurement. We found that the log of the ratio of R5a to P1 [log(R5a/P1)] and of R5b to P1 [log(R5b/P1)] were significantly (p < 0.0001) elevated in HCC rats, but not in cirrhotic, fibrotic and control animals (Figure 3). In contrast, P5 decreased significantly with development of fibrosis and progression to cirrhosis, but it did not decrease further with development of HCC (Figure 3). This indicates that P5 is associated with progression of fibrosis. On the basis of these results, we propose the use of the GlycoTest for monitoring pathology in DENA-treated rats, as depicted in Figure 4: monitoring fibrosis progression by decreases in P5 and HCC progression by increases in R5a and R5b, and detecting HCC by elevation of log(R5a/P1) or log(R5b/P1).

### Evaluation of serum glycan biomarkers after FTS treatment to prevent HCC

**Figure 5:**
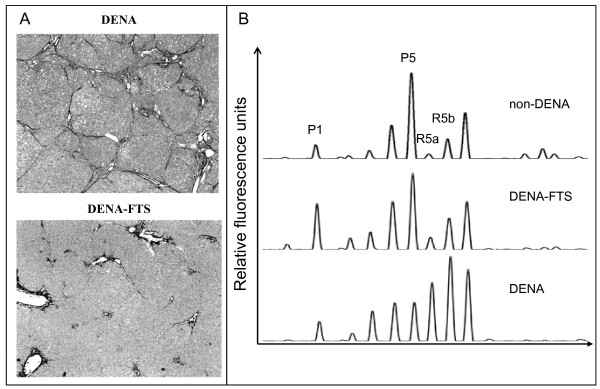
**Desialylated N-glycan profiles of serum proteins from DENA rats treated with FTS**. (A) Liver histology after 16 weeks of treatment with DENA or DENA plus FTS (DENA-FTS). Sirius staining shows that FTS treatment prevented development of cirrhosis. (B) The three groups of rats were the untreated control (top), DENA plus FTS (DENA-FTS) (middle), and DENA alone (bottom). The vertical axis represents the glycan values of the peaks as percent relative fluorescence level. The horizontal axis represents the retention time of N-glycans.

**Figure 6:**
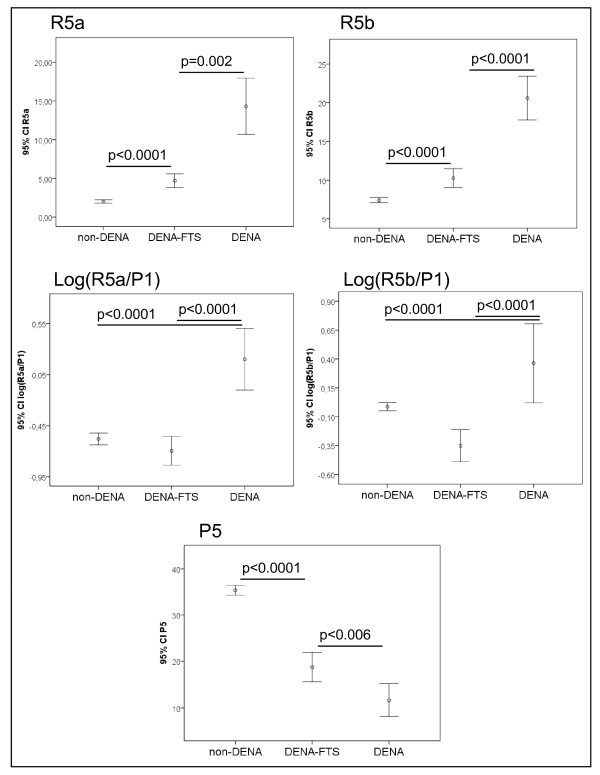
**The GlycoTest markers in DENA rats treated with FTS**. The levels of GlycoTest markers changed towards non-DENA values after FTS treatment. Error bars represent 95% confidence intervals for means. Statistical significance of differences between groups is indicated by the p value.

FTS has been shown to act as a functional Ras antagonist in cells. It acts mainly by competing with Ras-GTP for binding to specific binding sites in the plasma membrane, and thereby it prevents active Ras from activating intracellular downstream signaling pathways [22]. We previously showed that administration of DENA alone to rats for over 16 weeks leads to formation of numerous whitish nodules over the surface of the liver and that the nodules are histologically compatible with HCC [23]. Treatment of DENA-treated rat with FTS (DNA-FTS) for 11 weeks prevented formation of nodules and histological manifestations of HCC [23]. In the present study, we evaluated our GlycoTest model using glycan biomarkers in FTS treated DENA rats: FTS prevented development of cirrhosis, as confirmed macroscopically and histologically (Figure 5A). N-glycan profiles in sera from animals treated with DENA (n = 4) or DENA-FTS (n = 8) were analyzed (Figure 5B). We used the procedure outlined in figure 4 and found that serum values of P5, R5a and R5b changed towards non-DENA values after 11 weeks of FTS administration, which indicates that FTS treatment prevents development of cirrhosis (Figure 6). Likewise, in rats treated with FTS, the specific HCC biomarkers log(R5a/P1) and log(R5b/P1) reverted to levels seen in rats not treated with DENA, which indicates that FTS prevents formation of HCC (Figure 6).

### Increased level of core-α-1,6-fucosylation during progression of HCC

The N-glycan structures for the peaks in humans have been described [16]. Overlay of the rat profiles with human serum N-glycan profiles revealed that P1 (NGA2F), P2 (NGA2FB), P5 (NA2) and P6 (NA2F) migrated at the same rates as the corresponding human structures during the CE (Additional file 2, Figure S2). However, peaks R3, R4, R5a and R5b were not found in human serum, which indicates that N-glycosylation is somewhat species-specific. This was confirmed by mass spectrometric glycomics profiling of serum from control and DENA rats. As shown in Additional file 3, Figure S3 the rat serum contains some glycans whose antennae are terminated with alpha galactose moieties. These glycans cannot be biosynthesized in humans [24,25].

**Figure 7:**
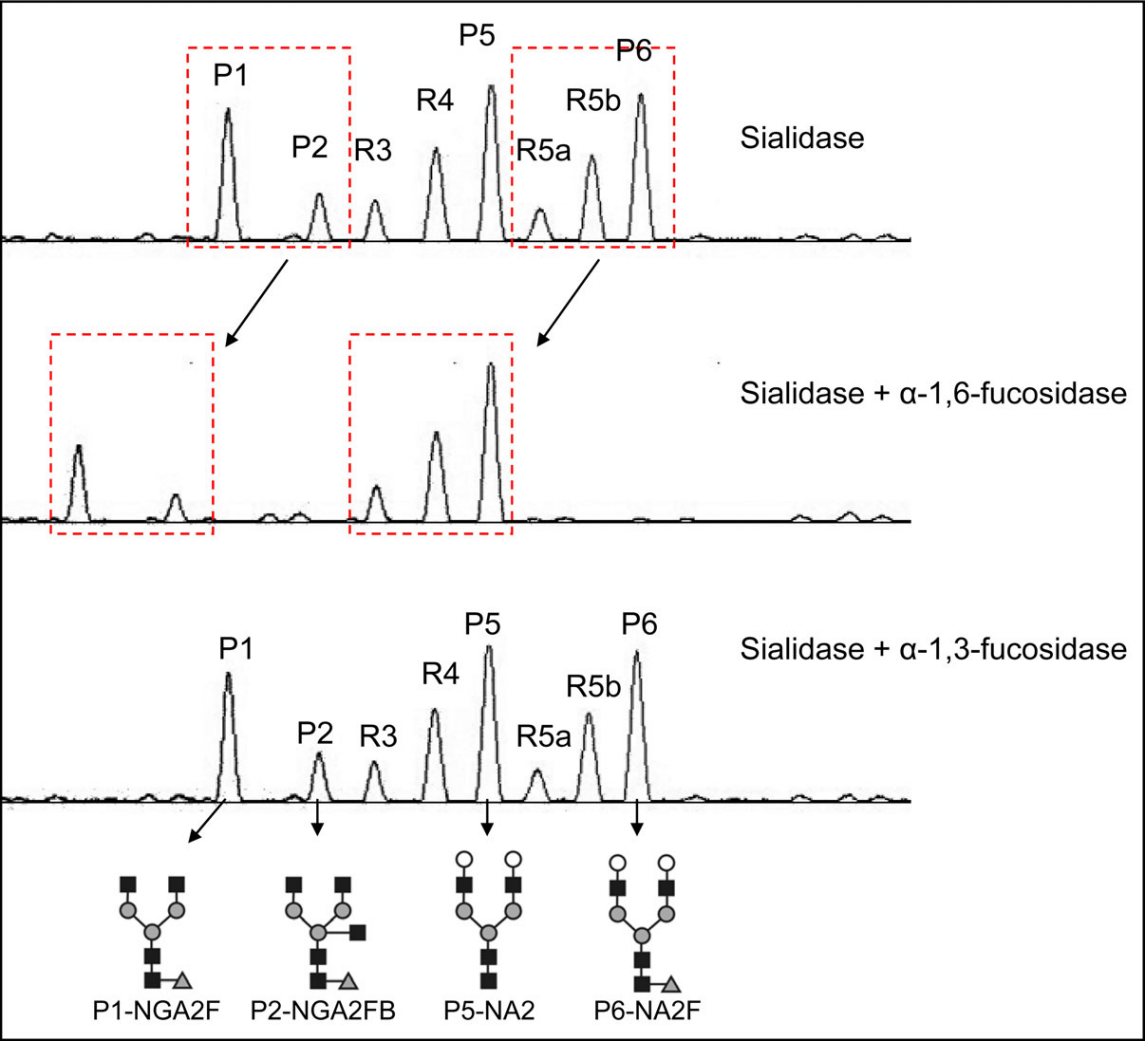
**Exoglycosidase sequencing of N-glycans from rat serum glycoproteins**. The upper panel shows separation of desialylated rat serum N-glycans: desialylated N-glycans digested with bovine kidney α-1,6-fucosidase (middle panel) and desialylated N-glycans digested with almond meal α-1,3-fucosidase (lower panel). Peaks P1, P2, R5a, R5b and P6 contain α-1,6-fucosylated structures. The arrows indicate the changes in the glycan peaks due to glycosidase digestion that are outlined in the dashed squares. The structures of the N-glycan peaks are shown below the panels. P1 is an asialo, agalacto, core-α-1,6-fucosylated biantennary glycan (NGA2F). P2 is an asialo, agalacto, core-α-1,6-fucosylated bisected biantennary (NGA2FB). P5 is an asialo, bigalacto, biantennary glycan (NA2). P6 is an asialo, bigalacto, core-α-1,6-fucosylated biantennary (NA2F). The symbols used in the structural formulas are the following: "black square" stands for N-acetylglucosamine (GlcNAc); "open circle" stands for galactose; "grey triangle" stands for α-1,6-linked fucose; "grey circle" stands for mannose.

**Figure 8:**
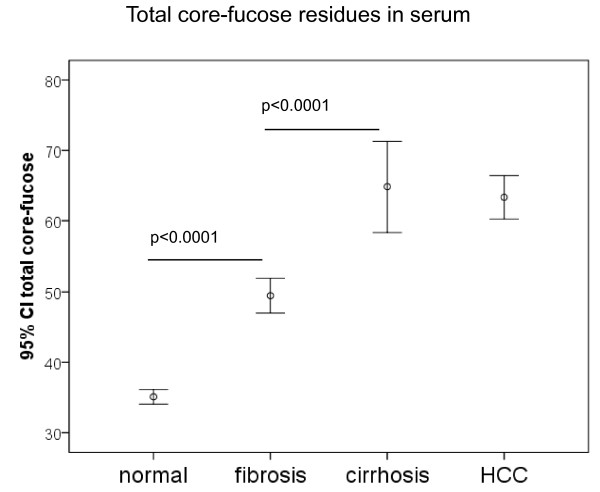
**Total serum core-fucose residues measured by DSA-FACE**. Error bars represent 95% confidence intervals for means. Statistical significance of differences between groups is indicated by the p value.

We studied the glycan structures using exoglycosidases following by DSA-FACE. We found that the structures of R5a and R5b were α-1,6-fucosylated, as they are shifted one residue forward after bovine kidney α-fucosidase digestion, which can remove only one α-1,6-fucose from the core N-acetylglucosamine; by contrast, R3 and R4 were not α-1,6-fucosylated (Figure 7). We did not detect branching α-1,3/4-fucosylated glycans at significant levels in rat serum because none of the peaks showed detectable changes after almond meal α-1,3/4-fucosidase digestion. All serum core-1,6-fucosylated glycans (peaks 1, 2, R5a, R5b and 6) were significantly higher in DENA-treated rats than in untreated rats (see Figure 3 and Additional file 1, Figure S1). Indeed, serum total core-α-1,6-fucosylated glycans gradually increased in DENA rats during progression of liver cirrhosis compared to control rats (Figure 8). This was confirmed by probing western blots of total serum proteins with lectin of *Aspergillus oryzae *(AOL), a specific carbohydrate-binding or carbohydrate-crosslinking protein that recognizes α-1,6-fucosylated glycans [26]. This showed that binding of serum glycoproteins was considerably increased in rats with cirrhosis or HCC compared to those with fibrosis and to control animals.

**Figure 9:**
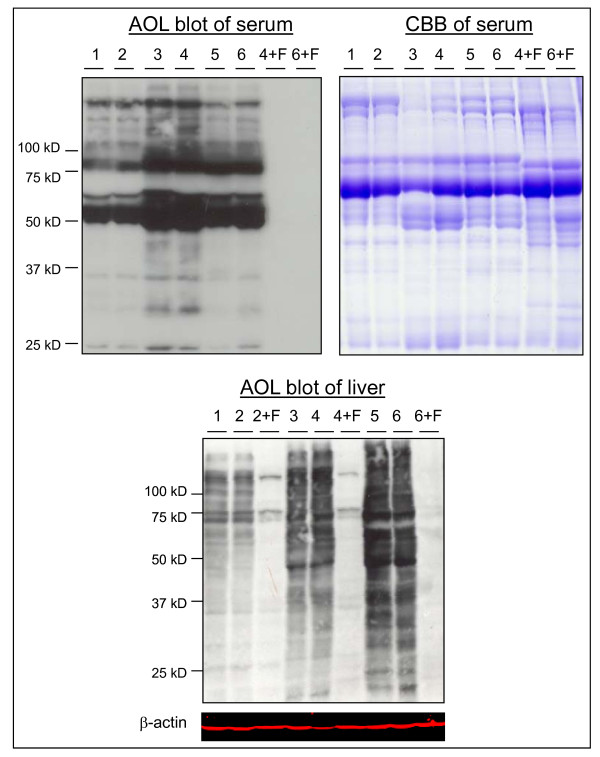
**SDS-PAGE of proteins from serum and liver and western blots probed with AOL**. The upper panel shows a western AOL blot of serum proteins (left) and a gel stained with Coomassie Blue (CBB) of the same serum proteins (right). The lower panel shows a western blot of liver proteins probed with AOL and β-actin. The lanes are as follows: 1-2: controls; 3-4: cirrhosis; 5-6: HCC. 2+F, 4+F and 6+F are the proteins in lanes 2, 4 and 6 digested with PNGase F and used as negative controls for AOL binding. The data were reproducible in three independent experiments.

To determine whether the increase in serum core-fucosylated glycoproteins was due to their secretion from the livers during progression of HCC, we probed blots of liver total proteins with AOL. This showed that the level of total core fucose residues was higher in the liver with cirrhosis and HCC than in control tissues (Figure 9).

### Alteration of the expression level of *α1,6-fucosyltransferase (FUT8) *in the liver of DENA rats

**Figure 10:**
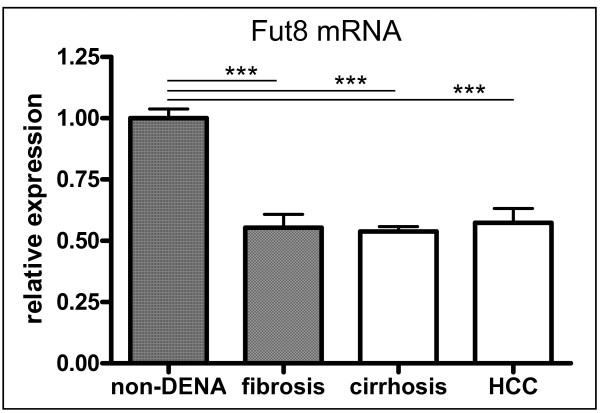
**The relative gene expression level of α-1,6-fucosyltransferase (FUT8) in the liver as measured by Q-PCR**. The horizontal axis represents the experimental groups: control (n = 6), fibrosis (n = 6), cirrhosis (n = 6) and HCC (n = 6). The vertical axis indicates the expression level of FUT8 relative to the mean of two housekeeping genes. Statistical significance of differences between groups is indicated by asterisks: *** p < 0.001.

The terminal glycosylation sequences produced by the cell are presumed to reflect the expression of the corresponding glycosyltransferase. Mammalian α1,6-fucosyltransferase (α1,6FucT), also called FUT8, catalyzes the transfer of a fucose residue from GDP-β-L-fucose, the donor substrate, to the innermost GlcNAc residue in *N*-glycan via an α1,6-linkage. To determine whether alteration of total core fucosylation in DENA rats was due to alteration of the glycosylation biosynthesis pathway, real-time PCR was used to analyze the expression of FUT8 in liver tissue of DENA-injected rats (HCC group and fibrosis/cirrhosis group) and non-DENA rats. To our surprise, *Fut8 *gene expression was much less in DENA rats than in control rats (Figure 10), which indicates that FUT8 is not responsible for the high level of core-1,6-fucosylation in DENA induced HCC.

## Discussion

Injection of DENA in rats led to development of cirrhosis followed by HCC. N-glycan analysis of rat serum showed that levels of N-glycans R5a and R5b were positively correlated with progression of fibrosis and HCC in DENA rats, whereas P1 (NGA2F) and P2 (NGA2FB) first increased with severity of fibrosis and then significantly dropped after development of HCC. Log(R5a/P1) and log(R5b/P1) were tumor specific markers that were significantly elevated in HCC but not in DENA-induced fibrosis and cirrhosis. Moreover, the value of P5 was negatively correlated with fibrosis degree, which makes it a specific marker for progression of liver cirrhosis in DENA rats. We propose that the GlycoTest model involving the above-mentioned serum glycan markers can be used for monitoring the progression of cirrhosis and HCC in the DENA rat model.

We also evaluated the GlycoTest model in DENA rats treated with the anticancer drug, FTS. A previous study demonstrated that administration of DENA leads to formation of numerous whitish HCC nodules over the surface of the liver as well as to the development of cirrhosis, and that FTS treatment prevents both lesions [23]. As expected, the GlycoTest markers for cirrhosis and HCC progression (P5, R5a and R5b) in the FTS treated rats changed towards non-DENA levels. This change correlated with the prevention of progression to cirrhosis and HCC that was confirmed macroscopically and by histology. The GlycoTest for HCC-specific markers, log(R5a/P1) and log(R5b/P1), completely reverted to non-DENA levels in the FTS treated DENA rats, thereby indicating complete prevention of HCC formation. Thus, we demonstrate that our GlycoTest model can be used as a non-invasive biomarker for monitoring progression of cirrhosis and HCC in DENA rats. The GlycoTest model can also be used to screen drug candidates for protective effects against HCC.

Increased levels of fucosylation have been reported in cancer, such as increased core-fucosylation of AFP in HCC [27,28], and increased fucosylation of haptoglobin in ovarian, lung, breast and pancreatic cancer [29,30]. Strong expression of α-1,6-FucT was observed in 3'-MeDAB-induced rat hepatomas and some rat hepatoma cell lines [31]. As recently reviewed by Miyoshi et al. [32], fucosylation is one of the most important types of sugar modification on glycoproteins in cancer. In agreement, our lectin blots showed increased fucosylation of N-glycans on total proteins in serum and liver in cirrhosis and HCC in the DENA rats. The total amounts of serum core-fucosylated structures were increased also during progression of HCC, partly due to alteration of the core fucosylated N-glycans of R5a, R5b, P1 (NGA2F), P2 (NGA2FB) and P 6 (NA2F). As a consequence, P5 (NA2), which is a precursor for core-fucosylation of biantennary glycan, was significantly decreased. N-glycans are synthesized in the ER and Golgi by the sequential addition of saccharides, such as fucose, by the corresponding glycosyltransferases [33,34]. Changes in the N-glycan profile in serum could be related to changes in the expression levels of glycosyltransferases in hepatocytes [33]. Basically, core-fucosylation is regulated by core-fucosyltransferase (FUT) and the GDP-L-fucose synthetic pathway. Based on sugar donor specificities, FUT transfers the substrate of GDP-L-fucose to the reducing terminal GlcNAc of the core structure of an asparagine-linked oligosaccharide. GDP-L-fucose is synthesized through a de novo pathway, a dominant pathway that involves conversion of cellular GDP-D-mannose to GDP-L-fucose by GDP-D-mannose-4,6-dehydratase (GMD) and GDP-4-keto-6-deoxy-D-mannose-3,5-epimerase-4-reductase (FX). Noda K et al. [35] described the relationship between elevated FX expression, increased production of GDP-L-fucose, and high levels of fucosylation in human hepatocellular carcinoma and hepatoma cell lines. Alpha-1,6*-*fucosyltransferase (FUT8) is the only fucosyltransferase involved in core-fucosylation in mice [36]. However, we did not detect increased expression of Fut8 gene in DENA rats, which might indicate that another yet unknown alpha-1,6-fucosyltransferase is involved. The mechanism behind the alteration of fucosylation during HCC development is not fully understood. It has been reported that overexpression of α-1,6-FucT in hepatoma cells suppressed intrahepatic metastasis after splenic injection in athymic mice, partly by a decrease in the adhesion of core fucosylated α5β1 integrin to fibronectin [37]. Recently, the function of core-fucosylation was associated with EGFR. The EGF-induced phosphorylation of EGFR is substantially blocked in Fut8 KO cells, and the binding of EGF to its receptor (EGFR) requires core fucosylation of N-glycans [38]. Diverse lines of evidence indicate that activation of the TGF-α/EGFR pathway contributes to HCC formation. It has been reported that the levels of TGF-α and EGFR mRNA increase at different stages of cirrhosis and HCC in the DENA rat model, and that blocking EGFR activity prevents development of HCC in DENA rats [19]. Increased core-fucosylation in HCC might be associated with enhanced core-fucosylation of EGFR, and thus with activation of EGF-induced phosphorylation of the EGFR pathway.

## Conclusions

We demonstrate that serum N-glycan markers can be used to monitor progression of HCC and to evaluate drugs in the DENA rat model. This GlycoTest model is particularly important because a rapid non-invasive diagnostic procedure for tumour progression in this rat model would greatly facilitate the search for anticancer drugs. Although the molecular mechanism by which N-glycosylation is altered in liver disease remains unsolved, our study confirms that abnormal fucosylation is important in HCC development, and that fucosylated glycoproteins could be exploited effectively for diagnosis of HCC in the DENA rat model.

## Methods

### Experiment design 1

Male Sprague-Dawley rats weighing 180-220 g were purchased from Shanghai SLAC Laboratory Animal Co., Ltd. and housed in cages under standard animal laboratory conditions in the experimental animal center of the Second Military Medical University. The rats (n = 60) were intraperitoneally injected with DENA at 20 mg/kg body weight three times a week for 14 weeks. The rats in the control group (n = 24) were injected with an equivalent volume of physiological saline at the same times. All animals were kept in a temperature controlled environment with a 12-h light-dark cycle and had free access to food and water throughout the study. To monitor tumor development, the rats were sacrificed at different times during and after DENA administration. Blood samples were collected for serum N-glycan analysis and several biochemical liver function tests. Liver tissues were fixed in 10% formalin for histological examination or frozen in liquid nitrogen and stored at -80°C for molecular biological study. All animal care and experimentation conformed to the guidelines established by the Second Military Medical University.

### Experiment design 2

It has been reported that trans-farnesylthiosalicyclic acid (FTS) prevents formation of DENA-induced HCC in rats [23]. Therefore, FTS-treated animals could be used as a validation group for HCC prevention. In brief, male Wistar rats (body weight 180-190 g) received weekly intraperitoneal injections of DENA at 50 mg/kg body weight for 16 weeks (DENA group). FTS was administered intraperitoneally at 5 mg/kg body weight three times a week starting six weeks after DENA induction (DENA-FTS group). The animals were sacrificed one week after the last DENA injection.

### Histology

The liver tissues were fixed with 10% formalin, embedded in paraffin, sectioned, and stained with hematoxylin and eosin (HE). Sirius red stain was used to detect fibrosis. Liver fibrosis was staged histologically by an experienced hepatopathologist using Scheuer's classification, which uses a scale from F0 to F4: F0, no fibrosis; F1, enlarged fibrotic portal tracts; F2, periportal or portal-portal septa, but intact architecture; F3, fibrosis with architectural distortion, but no obvious cirrhosis; F4, cirrhosis [39,40].

### Biochemical tests

Routine biochemical tests on sera, including total bilirubin (TBil), alanine aminotransferase activity (ALT), aspartate aminotransferase activity (AST) and γ-glutamyltransferase (GGT), were done using the standard methods (Hitachi 7600 Analyzer, Hitachi, Japan Wako Diagnostics reagents, Wako Pure Chemical Industries Ltd., Japan).

### N-glycan profiling using DSA-FACE

N-glycan profiles were generated from 2 μl serum samples by DSA-FACE technology by using a capillary electrophoresis (CE)-based ABI3130 Genetic Analyzer (Applied Biosystems, Foster City, CA, USA) as previously described [16,41]. Total N-linked glycans were released from serum proteins by digestion with peptide N-glycosidase F (PNGase F) (New England Biolabs, MA, USA), which cleaves most common mammalian N-linked oligosaccharides at the N-glycosidic linkage. N-glycans with reducing ends were derivatized with APTS (Molecular Probes, Eugene, OR, USA) and directly separated using the CE-based DNA sequencer ABI 3130. Because most serum glycans have a terminal sialic acid, which contains a negative charge, removal of sialic acid with hydrolyzing exoglycosidase (a sialidase from *Arthrobacter ureafaciens*) (Roche, Indianapolis, IN, USA) makes the glycan profile much clearer. The heights of the peaks were measured by GeneMapper version 3.7 software (Applied Biosystems), and the height of each peak was converted to a percent of the total of all peaks.

### Structure analysis of N-glycans using exoglycosidase digestion

One microliter of APTS-labeled desialylated N-glycan obtained from the DSA-FACE procedure was digested overnight with various mixtures of exoglycosidases in 10 mM NH_4_Ac (pH 5.0) at 37°C. The products were then subjected to electrophoresis on an ABI 3130. The exoglycosidases were α-1,3/4-Fucosidase (almond meal) (Glyko, Novato, CA, USA), which cleaves non-reducing terminal α-1,3/4-linked fucose, and α-1,6-Fucosidase (bovine kidney) (Glyko, Novato, CA, USA), which cleaves non-reducing terminal α-1,6-linked fucose.

#### MS and MS/MS Analyses of Permethylated Glycans

Approximately 1 *μ*L of rat serum sample was desialylated with hydrolyzing exoglycosidase (a sialidase from *Arthrobacter ureafaciens*) (Roche, Indianapolis, IN, USA). And then desialylated serum was subjected to reduction, carboxymethylation, and tryptic digestion: the sample was reduced in 1 ml of 50 mm Tris-HCl buffer, pH 8.5, containing 2 mg/ml dithiothreitol. Reduction was performed at 37°C in a water bath for 1 h. Carboxymethylation was carried out by the addition of iodoacetic acid (5-fold molar excess over dithiothreitol), and the reaction was allowed to proceed at room temperature in the dark for 1.5 h. Carboxymethylation was terminated by dialysis against 4 × 4.5 liters of 50 mm ammonium bicarbonate, pH 8.5, at 4°C for 48 h. After dialysis, the sample was lyophilized. The reduced carboxymethylated proteins were then digested with *N-p*-tosyl-l-phenylalanine chloromethyl ketone-pretreated bovine pancreas trypsin (EC 3.4.21.4; Sigma) for 16 h at 37°C in 50 mm ammonium bicarbonate buffer, pH 8.4. The products were purified by C_18 _Sep-Pak^® ^(Waters) as described previously [42].

Peptide *N*-glycosidase F digestion of the tryptic glycopeptides was carried out in 50 mM ammonium bicarbonate, pH 8.5, for 20 h at 37°C with 5 units of enzyme (Roche Applied Science, U.K.). The released *N*-glycans were purified from O-glycopeptides and peptides by chromatography on a Sep-Pak C18 cartridge (Waters Corp., Milford, MA) and subsequently methylated using the sodium hydroxide permethylation procedure as described previously [43].

MALDI-TOF data were acquired on a Voyager-DE STR mass spectrometer (Applied Biosystems, Foster City, CA) in the reflectron mode with delayed extraction. Permethylated samples were dissolved in 10 μl of 70% (v/v) aqueous methanol, and 1 μl of the dissolved sample was premixed with 1 μl of matrix (20 mg/ml 2,5-dihydroxybenzoic acid in 80% (v/v) aqueous methanol), spotted onto a target plate, and dried under vacuum.

Further MS/MS analyses of peaks observed in the MS spectra were carried out using a 4800 MALDI-TOF/TOF (Applied Biosystems) mass spectrometer in positive ion mode. Signals selected for MS/MS were (M + Na)+ molecular ions.

The collision energy was set to 1 kV, and argon was used as collision gas. Samples were dissolved in 10 *μ*L of methanol, and 1 *μ*L was mixed at a 1:1 ratio (v/v) with 2,5-dihydroxybenzoic acid (20 mg/mL in 70% methanol in water) as matrix.

The MS and MS/MS data were processed using Data Explorer 4.9 Software (Applied Biosystems, U.K.). The mass spectra were baseline corrected (default settings) and noise filtered (with correction factor of 0.7), and then converted to ASCII format. The processed spectra were then subjected to manual assignment and annotation with the aid of a glycobioinformatics tool known as GlycoWorkBench [44].

Peak picking was done manually, and proposed assignments for the selected peaks were based on molecular mass composition of the ^12^C isotope together with knowledge of the biosynthetic pathways. Sequence ambiguities occurring in proposed structures were resolved by data obtained from MS/MS experiments.

### Western lectin blot

A total of 25 μg serum proteins or 50 μg proteins extracted from frozen liver tissues were separated by electrophoresis in 10% sodium dodecyl sulfate-polyacrylamide gel. The proteins were then transferred to a nitrocellulose membrane for lectin blot analysis. The membranes were blocked overnight at 4°C with 3% bovine serum albumin (BSA) in Tris-buffered saline (140 mM NaCl, 10 mM Tris-HCl, TBS) and then incubated for 1 h at room temperature with 5 μg/ml of biotinylated *Aspergillus oryzae *L-fucose-specific lectin (AOL) (Funakoshi Co., Ltd., Tokyo, Japan) in TBST buffer (TBS containing 0.05% Tween 20). After four washes of 5 min each with TBST, the membranes were incubated with 1/1000 diluted horseradish peroxidase-conjugated Streptavidin (R&D Systems, Minneapolis, MN, USA) for 1 h at room temperature. The membranes were washed four times again with TBST and developed with an ECL system (Amersham Biosciences Inc., Piscataway, NJ, USA).

### RNA extraction and real-time RT-PCR

RNA was extracted from frozen liver tissues with Qiagen RNeasy mini kit according to the manufacturer's instructions (QIAGEN GmbH, Hilden, Germany). The purity and concentration of RNA were determined by a Nano Drop ND-1000 spectrophotometer (NanoDrop Technologies, Wilmington, DE, USA). cDNA was synthesized starting from 2 μg of total RNA by using iScript cDNA Synthesis Kit (Bio-Rad Laboratories Inc., Hercules, California, USA).

Real-time qPCR using the Light Cycler 480 (Roche Diagnostics, Vilvoorde, Belgium) was performed on a 16-fold dilution of the cDNA. Each 10-μl assay contained 5 μl of 2× Fast SYBR^®^ Green Master mix (Applied Biosystems, CA, USA) containing FastStart Taq DNA Polymerase, reaction buffer, dNTP mix (with dUTP instead of dTTP), SYBR Green I dye, MgCl_2_, 2 μl primer mix for each gene (final concentration of 0.5 μM each). To normalize the liver sample data, we used the genes for glyceraldehyde-3-phosphate dehydrogenase (GAPDH), tyrosine 3-monooxygenase/tryptophan 5-monooxygenase activation protein, and zeta polypeptide (Ywhaz). Each reaction was performed in triplicate. PCR cycling consisted of denaturation at 95°C for 5 min followed by 50 cycles of 95°C for 10 sec, 60°C for 30 sec, and detection for 1 sec at 72°C. The Ct data were analyzed with Excel and GraphPad Prism 4. Primers were designed using the Primer Express program (Applied Biosystems). The sequences are shown in Table 2.

**Table 2:** PCR primer pairs used in the present study

Rattus Norvegicus Gene	Forward Primer (5'-3')	Reverse Primer (5'-3')	Amplicon Length (bp)
FUT8	CCATCCTCGGCCTCCTTACT	ACTGGGACACCCACCACACT	101
GAPDH	ACTCTACCCACGGCAAGTTC	TACTCAGCACCAGCATCA	133
Ywhaz	AAAAAGGAGATGCAGCCGAC	GTTGAGGGCCAGACCCAGT	51

### Statistical Analysis

All quantitative variables are presented as mean ± SD. The unpaired t-test for comparison of two groups was used, and Welch's correction was applied if the assumption of equal variance was not accepted. For comparison of more than two groups, one-way ANOVA was performed. All reported p-values are two-tailed, and p-values < 0.05 are considered statistically significant. Statistical analysis was performed with SPSS 15.0 software (SPSS, Chicago, IL, USA). The coefficients of variation (CVs) for peak intensity were averaged in Microsoft Excel.

## Abbreviations

HCC: Hepatocellular carcinoma; DENA: diethylnitrosamine; DSA-FACE: DNA sequencer-assisted (DSA) and fluorophore-assisted carbohydrate electrophoresis (FACE); PNGase F: peptide N-glycosidase F; APTS: 8-amino-1,3,6-pyrenetrisulfonic acid; NA2: bigalacto biantennary glycan; NA2F: bigalacto core-α-1,6-fucosylated biantennary; FUT: fucosyltransferase; AOL: *Aspergillus oryzae *lectin; CBB: Coomassie Brilliant blue; FTS: farnesylthiosalicyclic acid;

## Competing interests

The authors declare that they have no competing interests.

## Authors' contributions

MF carried out the N-glycomics studies, data analysis, generated figures and wrote the paper. YZ carried out the sample collection, participated in the statistical analysis. YC, ML and BS performed DENA-rat experiment. XJ performed the biochemistry analysis. VV and SD performed N-glycomics studies. PS and YH carried out the animal studies with FTS and provided serum for the biomarker analyses. AD, SH and PG carried out MS and MS/MS analyses of permethylated Glycans. CL and CG carried out the experimental design and quality control. CC designed research, analyzed results, wrote and revised the paper. All authors read and approved the final manuscript.

## Supplementary Material

Additional file 1 **Figure S1**. Serum N-glycan values in the DENA rats with fibrosis, cirrhosis and HCC are shown. The vertical axis represents glycan values of P1, P2, R3, R4 and P6. Error bars represent 95% confidence intervals for means. Click here for file

Additional file 2 **Figure S2**. Serum desialylated N-glycan profiles from human and rat. The four panels from top to bottom are N-glycan fingerprint from human serum, N-glycan fingerprint from rat serum, N-glycan fingerprint from the pool sample of human and rat sera, and overlay of human and rat (blue curves represent a human profile and red curves represent rat profile). The vertical axis represents the glycan values of the peaks as percent relative fluorescence level. The X-axis represents the retention time of N-glycans. Click here for file

Additional file 3 **Figure S3**. Desialylated serum N-glycan structures were analysed by MALDI-TOF MS and MS/MS. S3-A: Profile of total permethylated N-glycans in tumor rat serum sample, S3-B: profile of total permethylated N-glycans in control rat serum. MALDI-MS/MS sequencing showed that the fucosylated components were mixtures. The majority are core fucosylated but a minor portion are antenna fucosylated. Click here for file
