# Supplementary figures and images for: Serum N-glycome biomarker for monitoring development of DENA-induced hepatocellular carcinoma in rat
Source: Mol Cancer. 2010 Aug 12;9:215. doi: 10.1186/1476-4598-9-215 (PMC2925372; doi:10.1186/1476-4598-9-215)

## Slide 1
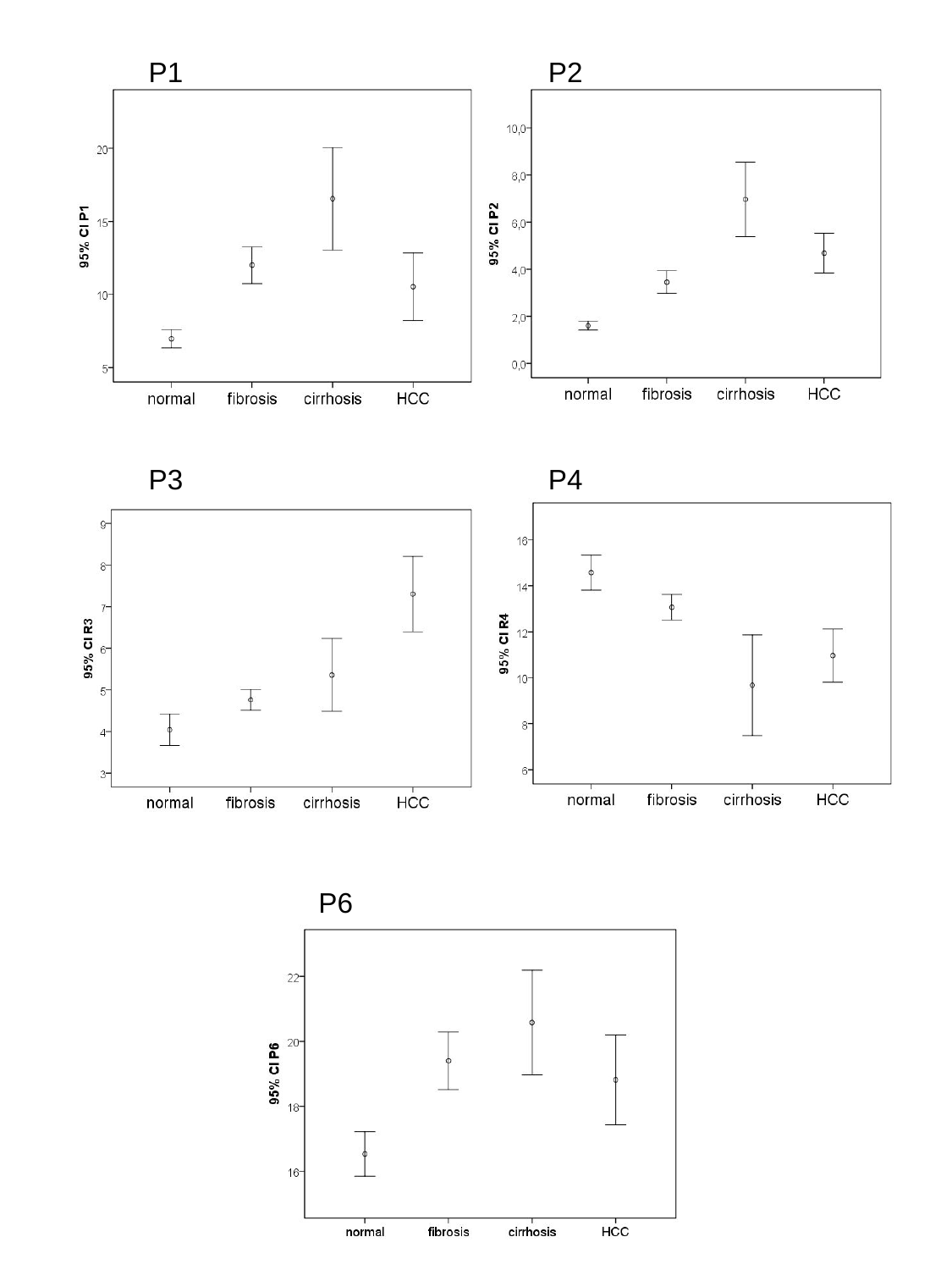

P1
P2
P3
P4
P6

Supplement: Additional file 1 — Figure S1. Serum N-glycan values in the DENA rats with fibrosis, cirrhosis and HCC are shown. The vertical axis represents glycan values of P1, P2, R3, R4 and P6. Error bars represent 95% confidence intervals for means. [file 1476-4598-9-215-S1.PPT]

## Slide 1
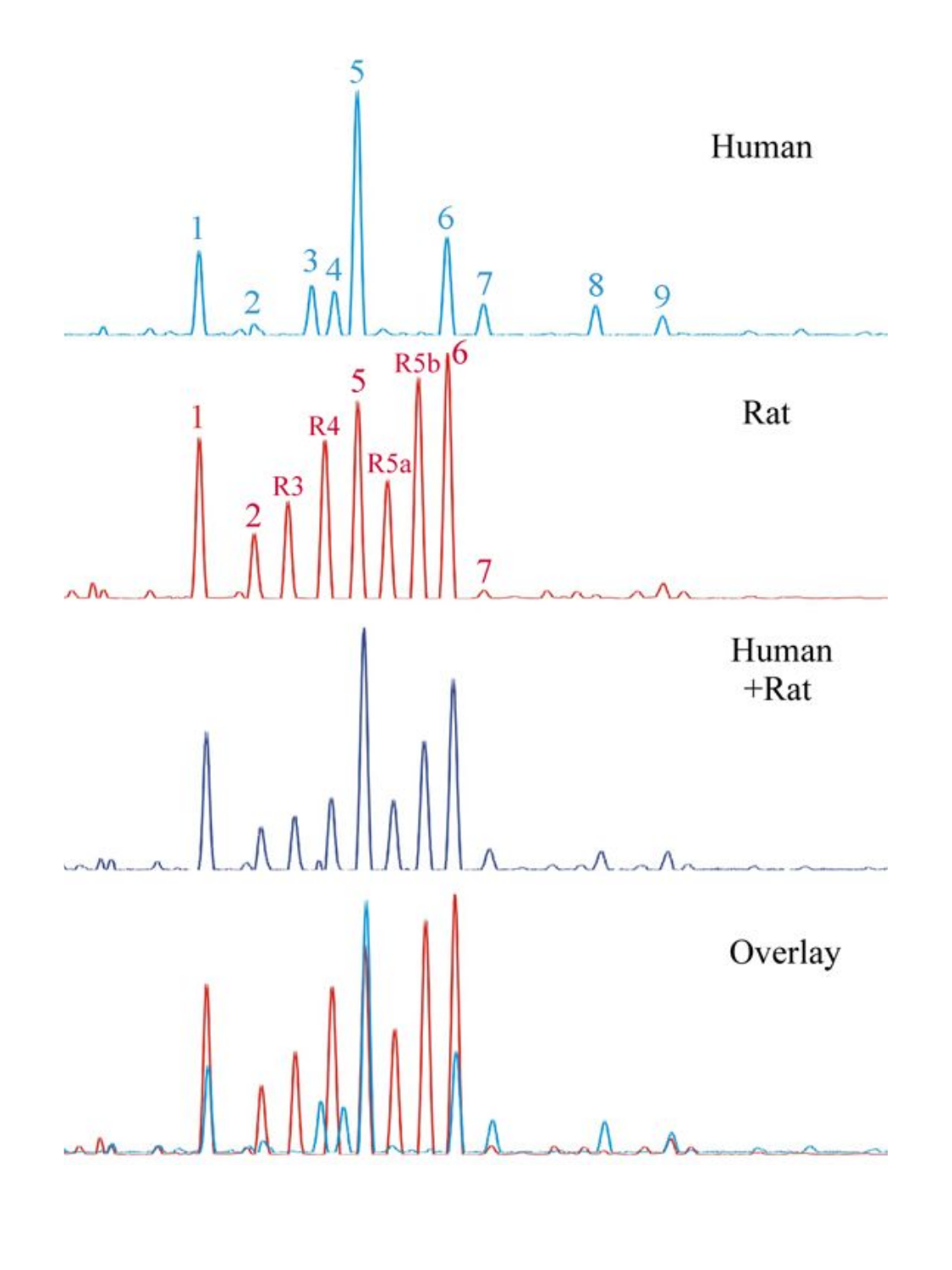

Supplement: Additional file 2 — Figure S2. Serum desialylated N-glycan profiles from human and rat. The four panels from top to bottom are N-glycan fingerprint from human serum, N-glycan fingerprint from rat serum, N-glycan fingerprint from the pool sample of human and rat sera, and overlay of human and rat (blue curves represent a human profile and red curves represent rat profile). The vertical axis represents the glycan values of the peaks as percent relative fluorescence level. The X-axis represents the retention time of N-glycans. [file 1476-4598-9-215-S2.PPT]

## Slide 1
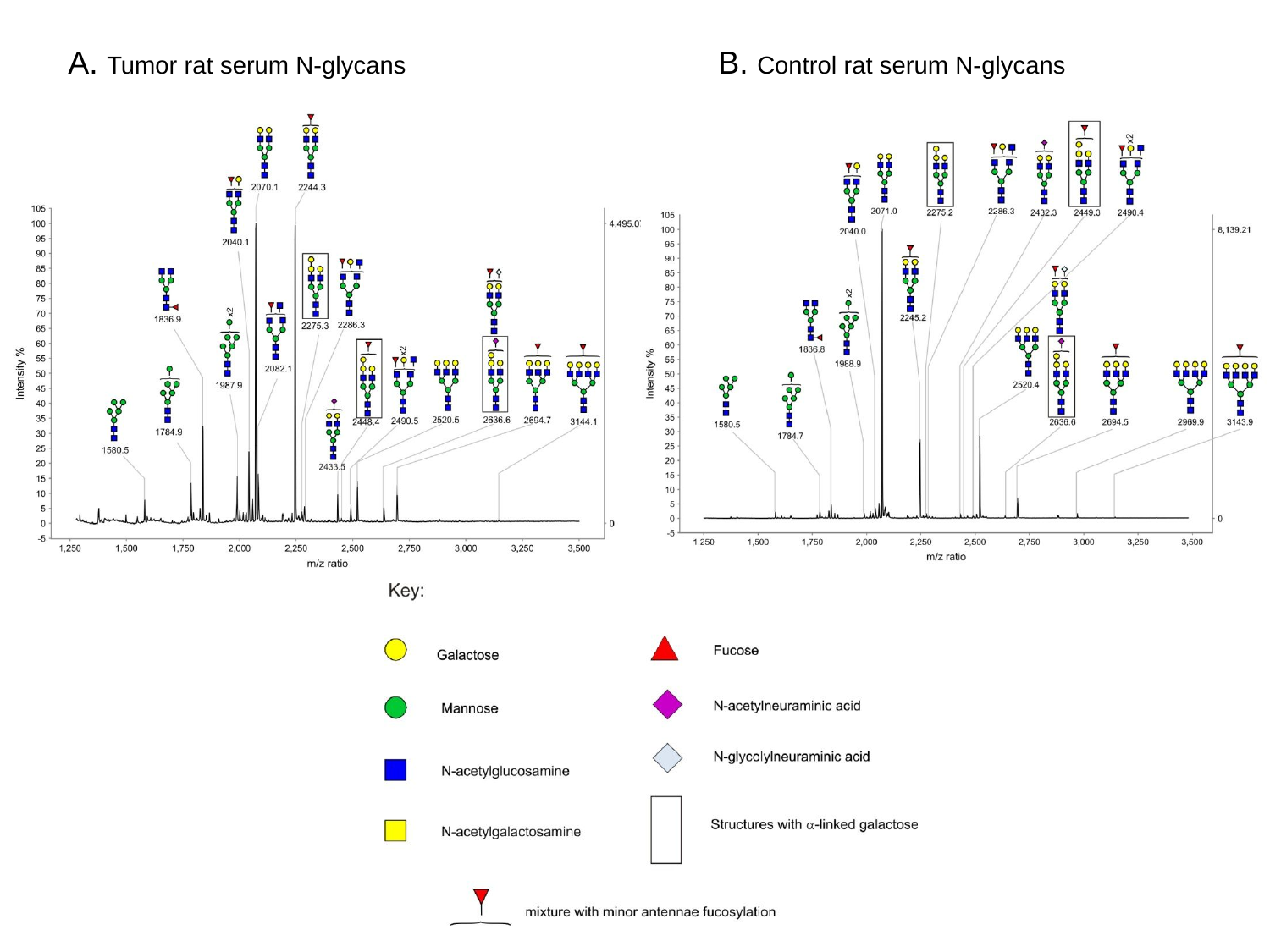

A. Tumor rat serum N-glycans
B. Control rat serum N-glycans

Supplement: Additional file 3 — Figure S3. Desialylated serum N-glycan structures were analysed by MALDI-TOF MS and MS/MS. S3-A: Profile of total permethylated N-glycans in tumor rat serum sample, S3-B: profile of total permethylated N-glycans in control rat serum. MALDI-MS/MS sequencing showed that the fucosylated components were mixtures. The majority are core fucosylated but a minor portion are antenna fucosylated. [file 1476-4598-9-215-S3.PPT]
